# Evaluation of the stiffness characteristics of rapid palatal expander screws

**DOI:** 10.1186/s40510-016-0151-z

**Published:** 2016-11-28

**Authors:** Luca Lombardo, Enrico Sacchi, Maria Larosa, Francesco Mollica, Valentina Mazzanti, Giorgio Alfredo Spedicato, Giuseppe Siciliani

**Affiliations:** 1 Postgraduate School of Orthodontics, University of Ferrara, Via Montebello 31, 44121 Ferrara, Italy; 2 Private Practice, Bologna, Italy; 3 Department of Engineering, University of Ferrara, Via Saragat 1, 44122 Ferrara, Italy; 4 UnipolSai Assicurazioni, Piazza della Costituzione 2, 40128 Bologna, Italy

## Abstract

**Background:**

The aim of this study is to evaluate the mechanical properties of the screws used for rapid expansion of the upper jaw.

**Methods:**

Ten types of expansion screw were assessed, seven with four arms: Lancer Philosophy 1, Dentaurum Hyrax Click Medium, Forestadent Anatomic Expander type “S”, Forestadent Anatomic Expander type “S” for narrow palates, Forestadent Memory, Leone A 2620-10 with telescopic guide, and Leone A 0630-10 with orthogonal arms; and three with two arms: Dentaurum Variety S.P., Target Baby REP Veltri, and Leone A 362113. A test expander with the mean dimensions taken from measurements on a sample of 100 expanders was constructed for each screw. The test expanders were connected to the supports of an Instron 4467 (Instron Corp., USA) mechanical testing machine equipped with a 500 N load cell, and the compression force exerted after each activation was measured. The mean forces expressed by the two- and four-arm expanders were then compared.

**Results:**

After five activations, the forces expressed by the two-arm devices were double than those expressed by the four-arm devices on average (224 ± 59.9 N vs. 103 ± 32.9 N), and such values remained high after subsequent activations.

**Conclusions:**

The expanders tested demonstrated stiffness characteristics compatible with opening of the palatine sutures in pre-adolescent patients. The stiffness of such devices can be further increased during the construction phase.

## Background

Normalizing the dimensions of the upper jaw is of primary importance in orthodontics. In fact, an upper jaw of incorrect dimensions may affect both the transversal and sagittal planes [1]. A rapid palatal expander (RPE) is the most popular device of choice in this regard, characterized by safety, predictability, and efficiency [2–8].

RPEs have a predominantly orthopedic action, although they do bring about a certain degree of dental expansion, in turn provoking labial inclination of the teeth. This effect becomes more pronounced as age advances, since an increase in the interdigitation at the palatine suture increases its resistance to opening and reduces the orthopedic effects in favor of dental effects [9–12].

Indeed, the change in skeletal transversal dimensions decreases from 50 % to roughly a third of the quantity of RPE screw activation after the pubertal growth peak in initial permanent dentition [13].

Rapid maxillary expansion treatment is able to induce more pronounced transverse craniofacial changes at the skeletal level before the peak in skeletal growth, and skeletal outcomes of greater magnitude and stability can be obtained when the expander is used before the pubertal growth spurt. When RME therapy is performed after the pubertal peak, on the other hand, transverse changes shift to the dentoalveolar level [14, 15].

Various models of screws and operating protocols have been suggested, both for achieving standard expansion and for activating the premaxillary sutures via alternate phases of expansion and contraction [16–19]. In order to open the median palatine suture to a sufficient degree and contemporaneously avoid a significant dentoalveolar response, RPEs must exert intense levels of force within a short time-frame. Hence, they must possess sufficient stiffness characteristics to enable them to exert such forces without deformation, so as to minimize the inclination of the teeth [20, 21]. Furthermore, the stiffness characteristics of an expander must be increased when a patient presents with a particularly deep palate [22].

In this regard, the use of miniscrews to stabilize RPEs seems to be helpful, especially in late adolescence, and is currently the focus of ongoing research [23, 24]. However, to date, available data is scarce. For example, Muchitsch et al. [25] analyzed only the mechanical characteristics of the arms of RPEs, while Camporesi et al. [26] analyzed the compressive forces developed at each activation of three types of expander screw.

With a view to reducing patient discomfort and facilitating oral hygiene procedures, manufacturers are developing and marketing increasingly less bulky, more streamlined RPEs [27, 28], and we set out to evaluate the stiffness characteristics of several such devices.

## Methods

The experiment evaluated 10 of the rapid expansion screws found on the market, all in medical-grade stainless steel (see Table 1 for details). Seven of the expansion screws had four retention arms and three had two retention arms. Each was welded to orthodontic bands and evaluated for overall stiffness, which comprised not only the stiffness of the screws themselves but also the resistance of the entire structure, including the welded joints. The deformation of the screws alone was not tested, as enormous forces would need to be measured.

**Table 1:** Screw characteristics

Two-arm screws	Max. expansion	Arm *∅* (mm)	Screw body size (mm)	Amount of expansion per activation (mm)	Lot no.
Dentaurum Variety S.P. two-arms	12	1.48	9.6 × 5 × 3	0.8	435299
Veltri Target baby REP	13	1.45	11 × 6 × 4.5	0.8	700032
Leone A 362113 two-arms	13	1.48	10 × 6 × 4.5	0.8	12032901
Four-arm screws	Max. expansion	Arm ∅ (mm)	Screw body size (mm)	Amount of expansion per activation (mm)	Lot n°
Lancer Philosophy 1	10	1.55	8 × 8 × 3.5	0.8	RPE OOO440
Dentaurum Hyrax Click Medium	10	1.48	10 × 11 × 4	0.8	435361
Forestadent Anatomic Exp. Type “S”	10	1.48	12 × 7 × 3.5	0.8	48297815
Forestadent Type “S” for narrow palates	10	1.48	12 × 7 × 3.5	0.8	7399006
Forestadent Memory	10	1.48	15 × 10 × 4	0.8	14957593
Leone A 2620-10 with telescopic guides	10	1.48	14 × 11 × 4	0.8	12122001
Leone A 0630-10 with orthogonal arms	10	1.48	10 × 6 × 4.5	0.8	13011601

### RPE construction

Each screw was used in the construction of an RPE modeled on average values derived from measurements made on 100 expanders constructed to fit 100 Caucasian patients (54 females and 46 males) aged between 8 and 13. All patients of the sample needed a RPE treatment. The patients already treated by orthodontics were excluded. This age range was chosen because the RPE allows favorable orthopedic changes and it is widely utilized by patients of this age.

The measurements made on these 100 RPEs were as follows: (1) length of the anterior arms comprising the screw body; (2) length of the posterior arms comprising the screw body; (3) bend angle of the anterior arms; and (4) bend angle of the posterior arms. A copper wire shaped to fit the morphology of the retention arms was used to perform these measurements, which were made using a goniometer (angular measurements) and gauge (linear measurements). All measurements were performed by the same operator, and the sample means calculated are reported in Table 2.

**Table 2:** Means of measures

1. Measurement	Kind of measure	Mean value
	Length of posterior arms (including screw body)	41.3 mm
	Length of anterior arms (including screw body)	32.5 mm
	Angle between screw body and posterior arms	146.2°
	Angle between screw body and anterior arms	144°
	Distance between anterior and posterior arms	19.8 mm

**Fig. 1:**
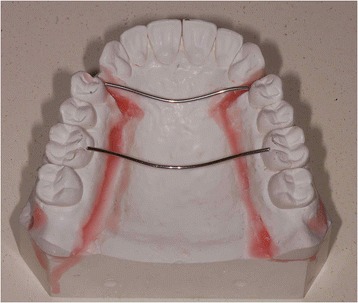
Transferring the mean values measured to the plaster models

Two metal wires (one for the anterior arms and one for the posterior arms) of 0.8-mm diameter were used to transfer these values to a plaster model of standard upper arch (Fig. 1). A set-up was performed to adapt the standard arch form to the means obtained from our measurements.

The first molars were removed from these models and replaced with analogous metal teeth, joined together by means of a threaded pin to ensure that they remained parallel and that the RPEs constructed around them would be correctly aligned with the mechanical testing machine; an Instron 4467 (Instron Corp., USA) with 500-N load cell was to be used for the stiffness testing.

**Fig. 2:**
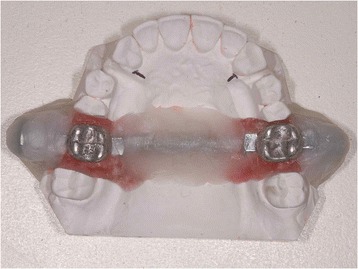
Master model featuring metal teeth

**Fig. 3:**
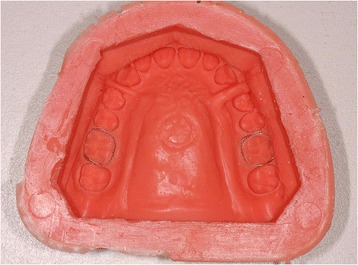
Duplication of the master model in silicone

Before testing, the metal teeth were fixed to the plaster model using wax to create a master model, duplicated for the construction of each test RPE to ensure that they all had identical form (Figs. 2 and 3). The parameters used in the construction of the test devices are also reported in Table 2.

Orthodontic bands (LEONE MOD. E8305 no. 14) were then fitted to the first molars of each model, and the test screw brazewelded on the bands. The RPE constructed for the four-arm screw featured palatal supports, and no bands on the premolars. The two-arm RPEs were welded to the first molars and featured no palatal supports. The constructed RPEs were bonded to the metal teeth using composite cement (ULTRA BAND-LOCK, Reliance, USA) and light-cured using an LED curing light (Elipar Freelight 2, 3M Espe, wavelength 430 ÷ 480 nm, intensity 1200 mW/mm^2^) for 30 s from the occlusal surface, the most effective method of bonding bands with this type of bonding agent [29].

### RPE activation procedure

**Fig. 4:**
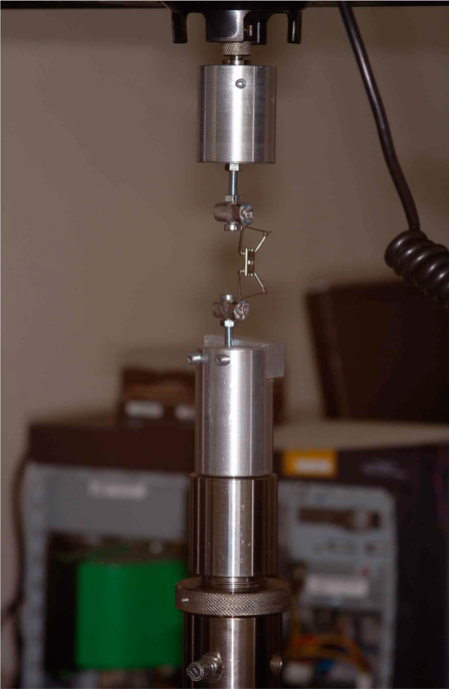
Test RPE attached to the Instron machine aligned with the major axis of the supports

The RPEs were fixed to the Instron machine via rigid supports connected to the metal teeth (Fig. 4). Each RPE was then activated by the key provided so that the point of application of the force on the teeth lay on the long axis of the support. The compression force expressed after each activation was measured until either the maximum separation capacity of the screw had been reached or the activation key had deformed. The means and standard deviations of the forces expressed by the two- and four-arm RPEs after the activations were calculated and compared.

### Statistical analysis

The statistical analysis aimed to assess how type (two arms, 2b, or four arms, 4b) and activation (1, 2,…, 26) influence the measured strength. It was performed on 10 unique models that were measured for up to 26 activations (the effective length of measurement varied by model) and the analysis was performed using the growth curve analysis [30] approach. In particular, the strength behavior upon activation was approximated using a three-degree polynomial as a function of activation (baseline model). Polynomial’s coefficients vary by type. To confirm the main conclusions *t*, a repeated measures ANOVA on the activation range where both 2A and 2B models were measured (1, 2,…, 11) consistently.

The polynomial functional expression of the baseline model is:

$$ strengt{h}_{it}={\alpha}_i+4{b}_i+\beta \times t+{\beta}_{4b}\times \mathrm{t}+\gamma \times {\mathrm{t}}^2+{\gamma}_{4b}\times {t}^2+\delta \times {t}^3+{\delta}_{4\mathrm{b}}\times {t}^3+{\upvarepsilon}_{\mathrm{it}} $$

Specifically, the strength values observed at time *t* on the *i*th model are as follows:

- A model-specific intercept (treated as random effect), indicating material specific strength response tendency
- A constant intercept that applies when the model is 4b type, indicating the baseline variation of strength due for the sample being of 4b type
- The terms *β*, *γ*, and *δ*, respectively, represent the first-, second-, and third-degree polynomial coefficients and are supplementary polynomial terms that applies when the model is 4b. The cubic curve tries to approximate the non-linear behavior of strength against activation, while the 4b terms represent interaction terms that allow the polynomial shape to change between 4a and 4b models which represent an additive error term. The repeated measures ANOVA assumed the type variable as the “between” factor and the activation (levels ranging from 1 to 11) as the “within” factor. A post hoc analysis has been therefore performed to compare difference in strength means between 4b and 2b types by activation level.

The statistical significance was assessed using a 5 % threshold. R software [31] was used throughout the data processing, and the lme4 R software package [32] was used to estimate both the growth curve and the repeated measures ANOVA. Post hoc analysis was performed using lsmeans R package [33].

## Results

**Fig. 5:**
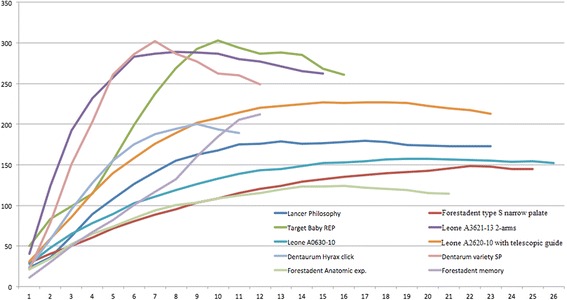
Results of the RPE stiffness tests

Figure 5 shows the results of the stiffness of the complete RPEs welded to the bands; the *x* axis shows the number of activations, and the *y* axis the force expressed in Newtons. The curves terminate at the point at which further activation was not possible due to screw block. The activation keys started to show signs of deformation around the 150-N mark. The greater the stiffness of the RPE (the slope of the activation/force curve), the greater the force at a particular activation, and as the graph shows, the two-arm RPEs present a far steeper curve than the four-arm devices and express considerable forces even from the initial activations, reaching maximum values of 288 N (Leone A3621-13), 302 N (Dentaurum Variety SP), and 303 N (Target Baby REP), respectively. In each case, after the peak, the deformation of the retention arms and screw body prevented further increases in force. In contrast, the force per activation of the four-arm screws was smaller, as seen by the flatter, more regular curves on the graph. In particular, the Forestadent Memory screw contains NiTi springs within the RPE body, which allows a more continuous, uniform expression of the force, reflected in the flatter curve up to the maximum force of 212 N, at which the screw blocks.

According to our results, the stiffest four-arm screw is the Leone A2620-10, which features telescopic guides, at a maximum force of 227 N, followed by the Dentaurum Hyrax Click, which expressed a maximum force of 200.2 N. The other RPEs present curves characterized by a quasi-linear trend up to the ninth or tenth activation, followed by a steady reduction down to a plateau at which the force remains almost constant as the activations progress. Among these, that which developed the highest maximum force was the Lancer Philosophy 1 (179.9 N), followed by the Leone A0630-10 with orthogonal arms (157.5 N), the Forestadent Type S for narrow palates (148.6 N), and, finally, the Forestadent Anatomic Expander (124 N).

**Fig. 6:**
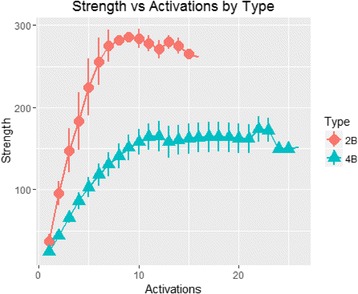
Comparison of two- and four-arm RPE stiffness

Table 3 and Fig. 6 show the means and standard deviations expressed by the two- and four-arm RPEs during activations.

**Table 3:** Results of stiffness tests

Type	Activations	Number	Mu	SD
2B	1	3	37	14.6
2B	2	3	96	24.7
2B	3	3	147	46.6
2B	4	3	183	61.3
2B	5	3	224	59.9
2B	6	3	256	49.7
2B	7	3	275	34.0
2B	8	3	282	11.0
2B	9	3	286	8.2
2B	10	3	284	20.7
2B	11	3	278	17.1
2B	12	3	271	19.7
2B	13	3	280	12.0
2B	14	3	275	14.1
2B	15	3	265	4.2
4B	1	7	24	6.6
4B	2	7	44	11.6
4B	3	7	66	18.0
4B	4	7	86	26.2
4B	5	7	103	32.9
4B	6	7	118	36.8
4B	7	7	131	39.2
4B	8	7	141	40.1
4B	9	7	151	41.6
4B	10	7	158	40.9
4B	11	7	164	42.2
4B	12	7	165	45.4
4B	13	7	158	42.8
4B	14	7	160	41.3
4B	15	7	162	41.5
4B	16	7	163	40.6
4B	17	7	164	41.2
4B	18	7	164	40.8
4B	19	7	164	40.4
4B	20	7	162	39.8
4B	21	7	162	38.6
4B	22	7	174	30.7
4B	23	7	172	29.2
4B	24	7	149	6.4
4B	25	7	149	6.8

The two-arm RPEs assessed in this study expressed more than double the force of their four-arm counterparts, even at five activations (224 ± 59.9 N vs. 103 ± 32.9 N), maintaining far higher values as the activations progressed. Indeed, after 10 and 15 activations, the force remained over 250 N, reflecting the conserved high level of stiffness. However, any more than 15 activations were prevented by structural deformations causing a block in the activation mechanism. In contrast, the four-arm RPEs continue to express a fairly constant force even after 20 activations, albeit at a much lower level.

The statistical analysis confirms the above descriptive considerations. The mixed-effect growth curve model coefficient estimates are shown in Table 4. In particular, the *t* (*t*) statistically greater of two in absolute value indicates significant effects, and the coefficient table shows that:

**Table 4:** Growth curve analysis coefficients

Term	Estimate	Std. error	Statistic	Significance
*α*	−47	21	−2.2	*
*β*	85	6.9	12	*
*γ*	−7.1	0.96	−7.4	*
*δ*	0.19	0.04	4.8	*
4*b*_*i*_	40	25	1.6	
*β*_4*b*_	−56	7.3	−7.7	*
*γ*_4b_	5.5	0.99	5.6	*
*δ*_4b_	−0.16	0.04	−4.1	*

The model was strength_*it*_ = *α*_*i*_ + 4*b*_*i*_ + *β* × *t* + *β*_4*b*_ × *t* + *γ* × *t* ^2^ + *γ*_4*b*_ × *t* ^2^ + *δ* × *t* ^3^ + *δ*_4*b*_ × *t* ^3^ + *ε*_*it*_

* = *p* value < 0.05

- The linear term *β* is positive, indicating an initial positive growth. The cubic term *γ* is negative indicating that the increase in strength levels off as far as activation progresses. The cubic term *δ* is negative finally. All the three terms are statistically significant indicating a non-linear behaviors of activation vs. strength.
- Activation and type 4b interaction (*β*_ab_) is negative and significant. This indicates that the initial increase is less steep for type 4b models.
- All the higher polynomial terms interaction are significant (*γ*_ab_, *δ*_ab_), indicating that the curvature for the growth dynamic of type 4b is different from that of the 2b models.

**Fig. 7:**
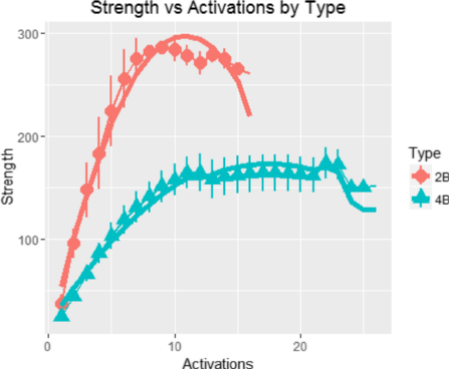
Strength dynamics of the two- and four-arm RPE

**Fig. 8:**
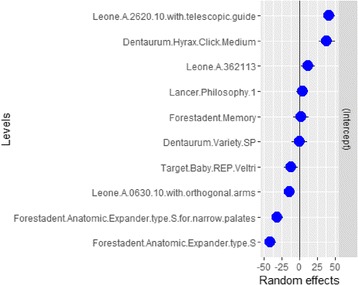
Intercepts of the two- and four-arm RPE

Figure 7 shows that a three-degree polynomial can well approximate the strength dynamics in the observed range. Growth curve model-specific intercepts (random effect) are shown in Table 5 and Fig. 8. A higher intercept indicates a higher starting level.

**Table 5:** Model-specific intercept

	(Intercept)
Dentaurum.Hyrax.Click.Medium	37
Dentaurum.Variety.SP	1.1
Forestadent.Anatomic.Expander.type.S	−39
Forestadent.Anatomic.Expander.type.S.for.narrow.palates	−31
Forestadent.Memory	0.27
Lancer.Philosophy.1	5.4
Leone.A.0630.10.with.orthogonal.arms	−16
Leone.A.2620.10.with.telescopic.guide	44
Leone.A.362113	13
Target.Baby.REP.Veltri	−14

**Fig. 9:**
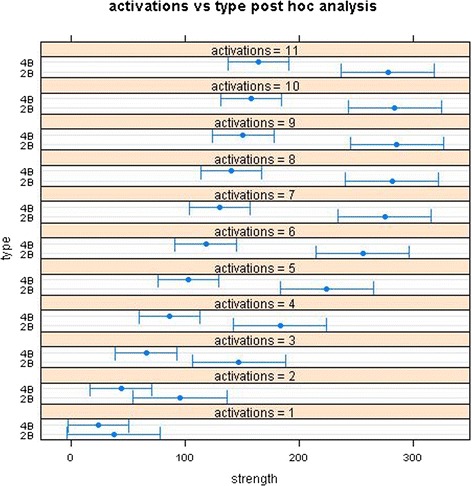
Post hoc repeated measures ANOVA

Post hoc means comparison analysis was carried on the repeated measures ANOVA used to confirm growth curve analysis results. Figure 9 displays graphically the results showing that 2b models consistently shows higher strength than 4b ones from activations greater than 2.

During the course of the experiments, no breakage or deformation of any of the bands associated with any of the RPEs tested occurred.

## Discussion

**Fig. 10:**
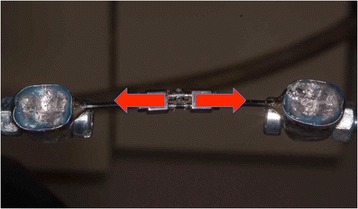
Two-arm RPEs: no bending moment on the horizontal plane

**Fig. 11:**
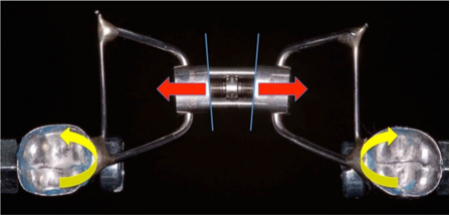
Four-arm RPEs: bending moment generated on the horizontal plane

**Fig. 12:**
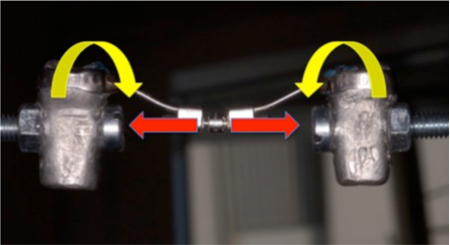
Two-arm RPEs: bending moment generated on the vertical plane

**Fig. 13:**
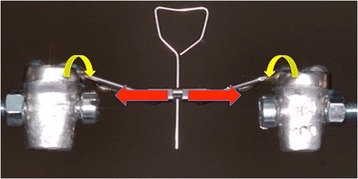
Four-arm RPEs: bending moment generated on the vertical plane

As shown in Figs. 10, 11, 12, and 13, the bending moments generated by the RPE arms were analyzed on both the horizontal and vertical planes. The greater stiffness of the two-arm RPEs may be linked to their smaller size, which allows them to be positioned on the same axis as the crowns of the molars used for anchorage. On the horizontal plane, this permits a point of application of the force to pass through the center of resistance of the system (which is determined by the expander as a whole, welded to the orthodontic bands), namely the anchoring teeth on which the bands are cemented. Hence, the mechanical stresses have an optimal distribution on the structure of the screw, without the generation of bending moment (Fig. 10). This differs from the four-arm RPEs, whose screw position creates bending moment that tend to deform the screw body on the horizontal plane (Fig. 11). Such deformations increase with the number of activations, both due to the increase in force applied and the resulting lengthening of the screw, which reduces its stiffness.

On the vertical plane, the line of force expressed by the screw is at the same distance from the center of resistance in the two types of RPEs. However, the two-arm devices are susceptible to greater deformation as they have two fewer arms and lack the palatal support that opposes vertical deformation (Figs. 12 and 13). This deformation involves both the screw body and the retention arms and is responsible for the deterioration in the force expressed by the two-arm RPEs after the peak.

**Fig. 14:**
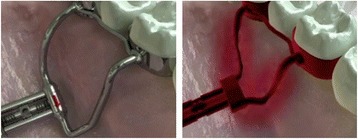
Difference between RPEs with arms perpendicular and parallel to the guides

Within the four-arm category, the differences between those that express greater and lesser forces may be linked to the design of the arms, which are parallel to the guide in the former category and perpendicular in the latter. In this latter category, the arms are longer and have greater bends, which reduces their stiffness and therefore the force expressed (Fig. 14).

Few studies on this topic can be found in the literature. In a recent study, Muchitsch et al. [25] compared the stiffness of the retention arms of 16 commercially available RPE screws and found a difference in stiffness of 37.32 %. Camporesi et al., [26] on the other hand, measured the force expressed by three different four-arm RPEs, like us using the Instron machine, but without any bands or welding; they revealed maximum forces ranging between 215 and 156 N. In contrast, Zimring and Isaacson [21], using intraoral dynamometers fixed to the RPEs mounted in the mouth of a sample of 5 patients, found forces ranging from 73 and 154 N. Sander et al. [34] also found smaller forces, between 70 and 120 N, needed to activate a special pre-calibrated screw mounted in ten 9- to 13-year-old patients via a hyper-rapid activation protocol (one or two activations, five times a day).

As regards comparison of two- and four-arm types of RPEs, Lamparsky et al. [27] conducted a study to evaluate the difference in their clinical effects, using radiographs to quantify the separation of the median suture and plaster models to measure the inter-canine distance, inter-molar distance, and arch perimeter before expansion, after the active expansion phase and after removal of the RPE. Their results suggest that there is a little difference in the clinical effects on the median suture and teeth brought about by two- and four-arm RPEs.

In light of these studies, our in vitro results show that the forces expressed by RPEs welded to anchorage bands appear sufficient to separate the median palatine suture in pre-adolescent and adolescent patients, although the force expressed by certain models may be insufficient for this purpose in older patients. In particular, models Forestadent type S for narrow palates and Forestadent Anatomic Expander generate low maximum forces (respectively, 148.6 and 124 N) that may be insufficient for the clinical demands reported elsewhere (Sander et al. [34]: 70–120 N, Zimring and Isaacson [21]: 73–154 N). That being said, it is important to note that the stiffness in vivo will be strongly influenced by clinical factors such as the stiffness of the median palatine suture and the circummaxillary sutures, which is far lower than that of the Instron machine.

Recent literature is ever more frequently proposing the use of RPEs in adult patients, or using protocols of alternating expansion and contraction, which severely test the mechanical resistance of such devices [16–19]. Hence, manufacturers should take into account the mechanical stiffness of the RPEs being manufactured, as well as comfort, hygiene, and versatility issues. RPEs need to be manufactured in such a way as to maximize skeletal effects and minimize unwanted dentoalveolar effects. That being said, the two-arm models we tested showed a loss of force due to deformation way beyond the levels of force clinically required to separate the median palatine suture and therefore appear to be fit for purpose in terms of stiffness, in agreement with the findings of the clinical trial conducted by Bratu et al. [28].

## Conclusions

The two-arm RPEs seem to be stiffer than their four-arm counterparts, and although from a mechanical perspective both are effective means of bringing about rapid expansion of the palate, certain models of the four-arm RPE may not express sufficient force to separate the median suture after puberty. The stiffness of the four-arm RPEs can be increased by using bands or bonding to fix them to the anterior teeth, but in addition to patient comfort, manufacturers should focus on the stiffness of both the retention arms and the screw body, whose propensity to generate a bending moment can be reduced by reducing its size. To this end, more research is needed into the resistance characteristics of RPEs, in particular to assess their suitability for older patients and alternating activation/contraction protocols and to determine how best to enhance skeletal and reduce unwanted dentoalveolar effects.
